# Description of the Use of Incentives and Penalties for Point-of-Care Ultrasound Documentation Compliance in an Academic Emergency Department

**DOI:** 10.7759/cureus.16199

**Published:** 2021-07-05

**Authors:** Myles Melton, Jordan D Rupp, Marc I Blatt, Jeremy S Boyd, Tyler W Barrett, Matthew Swarm, Michael J Ward

**Affiliations:** 1 Emergency Medicine, Olympia Emergency Services, Olympia, USA; 2 Emergency Medicine, Vanderbilt University Medical Center, Nashville, USA; 3 School of Medicine, Vanderbilt University, Nashville, USA

**Keywords:** point-of-care ultrasound, documentation, financial incentives, documentation workflow, emergency medicine

## Abstract

Objectives

Incomplete documentation and submission to the electronic health record of performed point-of-care ultrasound (POCUS) studies is problematic from a patient care, medicolegal, and billing standpoint. Positive and negative financial incentives may be used to motivate physicians to complete documentation workflow. The most efficacious route to improve POCUS workflow completion remains to be determined.

Materials and methods

A retrospective analysis of POCUS documentation in an academic emergency department during four distinct six-month blocks was performed. POCUS workflow completion was assessed without incentives (Baseline), with financial bonus (Incentive), interim period (Washout), and with a negative financial incentive (Penalty) to determine the effect of these incentives on workflow completion.

Results

There was an appreciable increase in the rate of POCUS studies documented between the “Baseline” (no incentive) and “Incentive” (small financial bonus) time periods. The improvement remained stable during the “Washout” (interim) period, and then increased further in the “Penalty” (negative financial incentive) period. This improvement was relatively diffuse among the providers studied. A similar pattern - improvements in the Incentive and Penalty periods with stability in the Washout - was also observed in the POCUS volume data (number of studies performed).

Conclusions

This study reveals a positive association between the implementation of both financial incentives and financial penalties, which increases in POCUS documentation among attending physicians at an academic emergency department.

## Introduction

Since the translation gap between research and practice is often cited as 17 years, strategies to enhance physician adoption of new technological practices have become the subject of much research [1]. Many studies have demonstrated benefits for emergency department (ED) patients when point-of-care ultrasound (POCUS) is used [2-4]. However, there appears to be variable adoption of POCUS use into clinical practice among emergency physicians [5]. The medical literature records the outcomes of some strategies to enhance POCUS use in emergency medicine [6,7]. These studies are few in number, limited in scope, and have been unable to indicate which strategies are the most reliably effective.

POCUS use is defined as a physician using ultrasound to answer a clinical question for patient care. The number of times resident and faculty physicians utilize ultrasound is recorded as POCUS studies performed. POCUS documentation refers to a clinically indicated ultrasound being performed and interpreted by the physician resulting in the images and the interpretation being properly uploaded to the electronic health record (EHR). The POCUS documentation rate is recorded as ultrasound studies submitted.

Beyond simply encouraging use, incomplete documentation of completed POCUS studies presents an additional challenge. Future providers are unable to access the collected data, negatively affecting patient care. It can also be problematic from a medicolegal standpoint as clinical decisions may be made with non-reproducible information. Finally, the Centers for Medicare & Medicaid Services require documentation - of indication, organs imaged, and interpretation(s) - in order to bill for any imaging study.

There is some evidence that financial incentives may be a favored reward for POCUS use and documentation among physicians, though enhanced education and training have also been described as beneficial interventions [8,9]. The most efficacious route to enhance physician use and documentation of POCUS remains to be determined.

## Materials and methods

This study was approved by the Institutional Review Board (ID number 200431).

Setting

**Figure 1:**
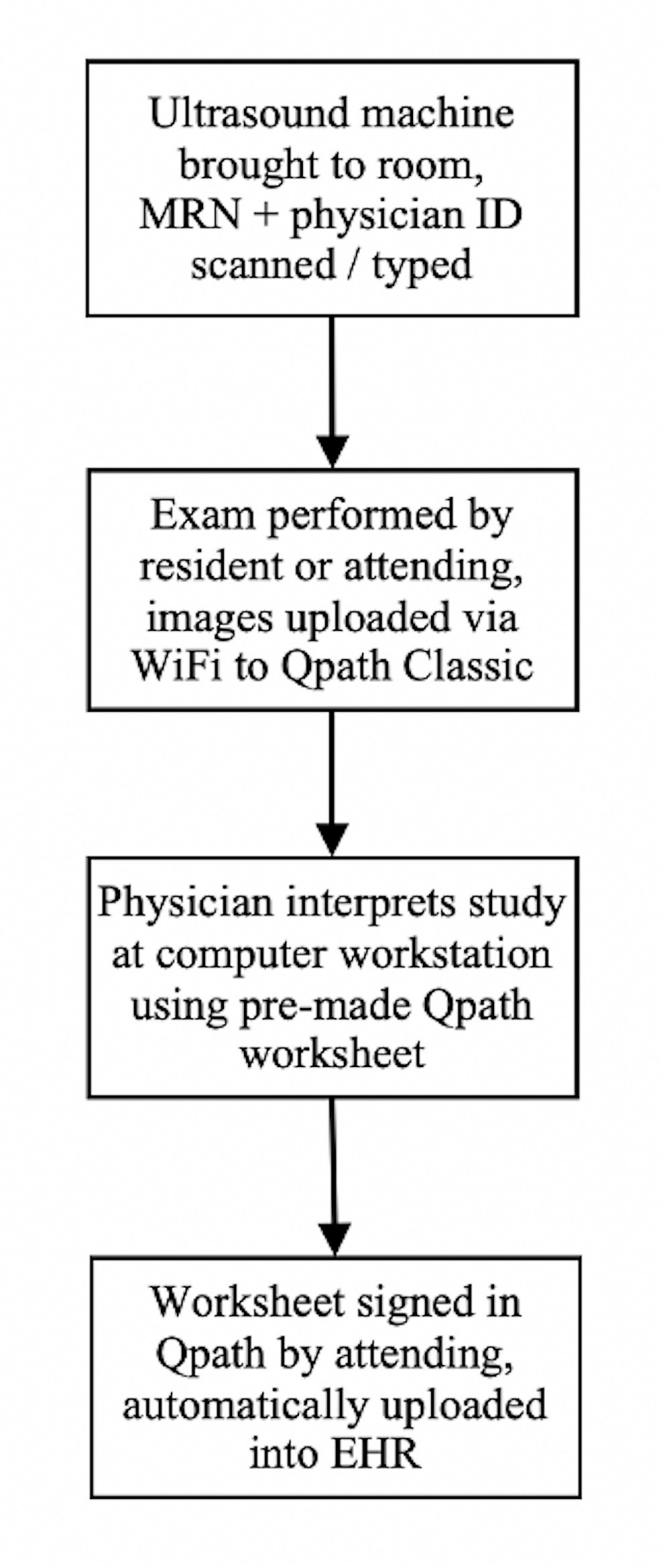
Outline of workflow for POCUS study submission. EHR, electronic health record; POCUS: point-of-care ultrasound.

The study institution is a tertiary-care academic ED with a Level 1 trauma designation that sees 70,000 patients annually. POCUS is taught to the residents in the department’s three-year residency program with 13 trainees in each year. There are 60 faculty members with varying levels of POCUS experience. Each year, all emergency medicine first-year residents complete a four-week rotation in POCUS. Additional dedicated training occurs over a four-week period for the second-year residents. The ED's access to ultrasound machines is as outlined in Table 1. The documentation workflow for POCUS has not changed significantly over the study period (Figure 1). Qpath “Classic” (Telexy Healthcare Inc., Maple Ridge, BC, Canada) is used to archive POCUS studies and Epic (Verona, WI, USA) is the EHR at the study institution.

**Table 1:** Description of ultrasound machine allotment and use.

ED Pod	Machine	Use
A	Mindray M9	General
A	SonoSite M Turbo	Intravenous Access
A	SonoSite X-Porte	Trauma Resuscitation
B/C	Mindray M9	General
B/C	SonoSite X-Porte	General

Participants

Only attending physicians employed by the study institution for the entire study period (January 2018-December 2019) were included in the analysis. In addition, six faculty members who were part of the Ultrasound Division were excluded as their ultrasound use and submission patterns are subject to many other forces outside of the incentives being evaluated. The Ultrasound Division is made up of faculty members who have all received fellowship-level POCUS training.

Design

Data collected throughout the study period were retrospectively analyzed. The 24 months under study were split into four distinct six-month blocks: January 2018-June 2018 (“Baseline” - no incentives applied); July 2018-December 2018 (“Incentive” - small financial bonus applied for physicians who appropriately completed the workflow and documented the ultrasounds performed during their shift - an average of one ultrasound submitted per shift); January 2019-June 2019 (“Washout” - an interim period between incentives); July 2019-December 2019 (“Penalty” - a portion of annual pay was withheld if physicians did not complete the workflow and document a minimum number of clinically performed ultrasounds).

Outcome measures

We measured the overall rate of POCUS use and documentation among the attending physicians at the study institution by using QPath Classic and EHR data. For each of the four time periods for each included attending physician, we recorded the number of ultrasound studies in QPath, both those with completed documentation resulting in submission to the Epic EHR and those with images acquired but not submitted. We define the number of “completed” studies as the number of POCUS studies performed by each attending physician. To collect these data, QPath was filtered to show only the studies with the specific attending listed as the attending of record. Then, the data were filtered for the time period of interest. If the attending physician completed the interpretation and signed the study, that study would be marked as having been “submitted” to the chart. When a study is reviewed and signed in this workflow, a document with images, video clips, and the interpretation report is generated in the patient’s chart in the EHR. The number of submitted studies for each attending over each time period was also recorded. Finally, to measure the number of patients seen in the ED, the number of patients for which that attending was listed as the attending of record was extracted and anonymized on a monthly level over the 24 months of the study.

Statistical methods

Results will be reported as ratios of studies submitted versus studies performed. This will be reported for the entire group of physicians and separated into performance quartiles based on the number of ultrasound studies performed. These results will be adjusted by simple proportions to reflect an ED with 100,000 patient visits per year so the results will be more easily applied to other institutions. Additionally, the number of studies per patient seen will be reported graphically, and individual provider submission rates per patient seen in each time period will be reflected in a heat map.

## Results

**Figure 2:**
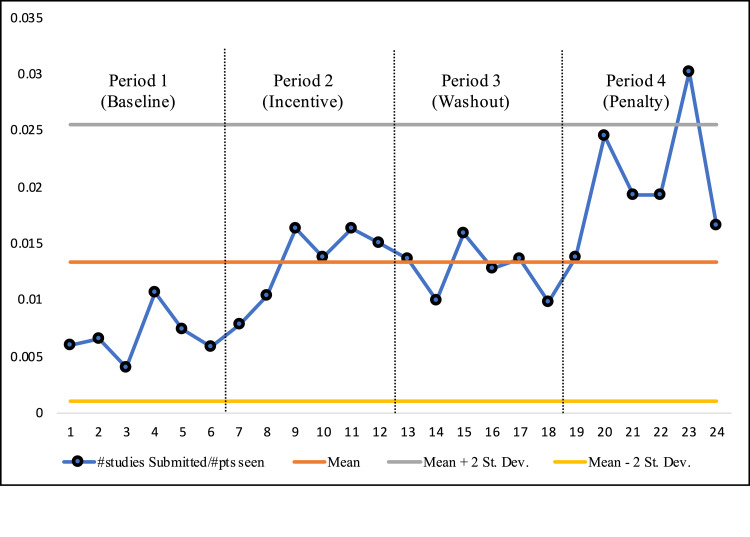
The number of studies submitted per patient seen for all providers combined over the study period (January 2018-December 2019) with each month labeled 1-24.

**Figure 3:**
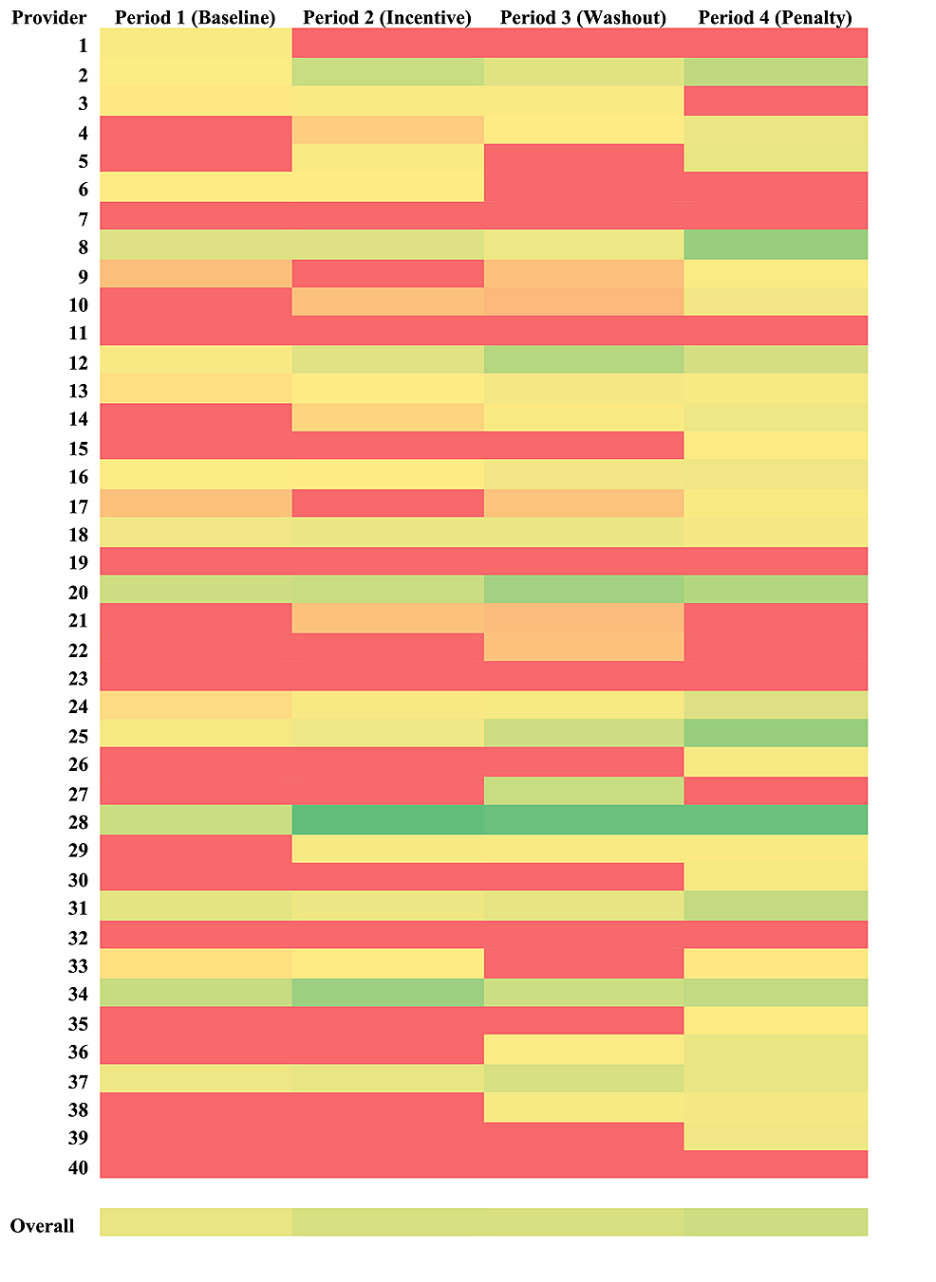
Provider heat map. Ultrasound studies submitted per patient seen for each provider in each time period. Red = low; Green = high.

The number of POCUS studies submitted to the EHR divided by the number of patients seen for each month is shown in Figure 2. There was an appreciable increase in this rate of ultrasound studies submitted per patient seen between the Baseline and Incentive time periods. This change remained stable during the Washout period. Finally, there was a second increase during the Penalty period. To analyze whether or not these increases during the Incentive and Penalty time periods were limited to specific providers, a heat map was created (Figure 3). For the 40 providers included in the study, the normalized documentation rate of ultrasound studies submitted per patient seen was compared over each of the four time periods with red demarcating relatively low rates and green representing relatively high rates. Looking at the heat map, it is clear that the improvements in documentation rate were spread diffusely among the providers studied rather than restricted to a small number of providers.

**Figure 4:**
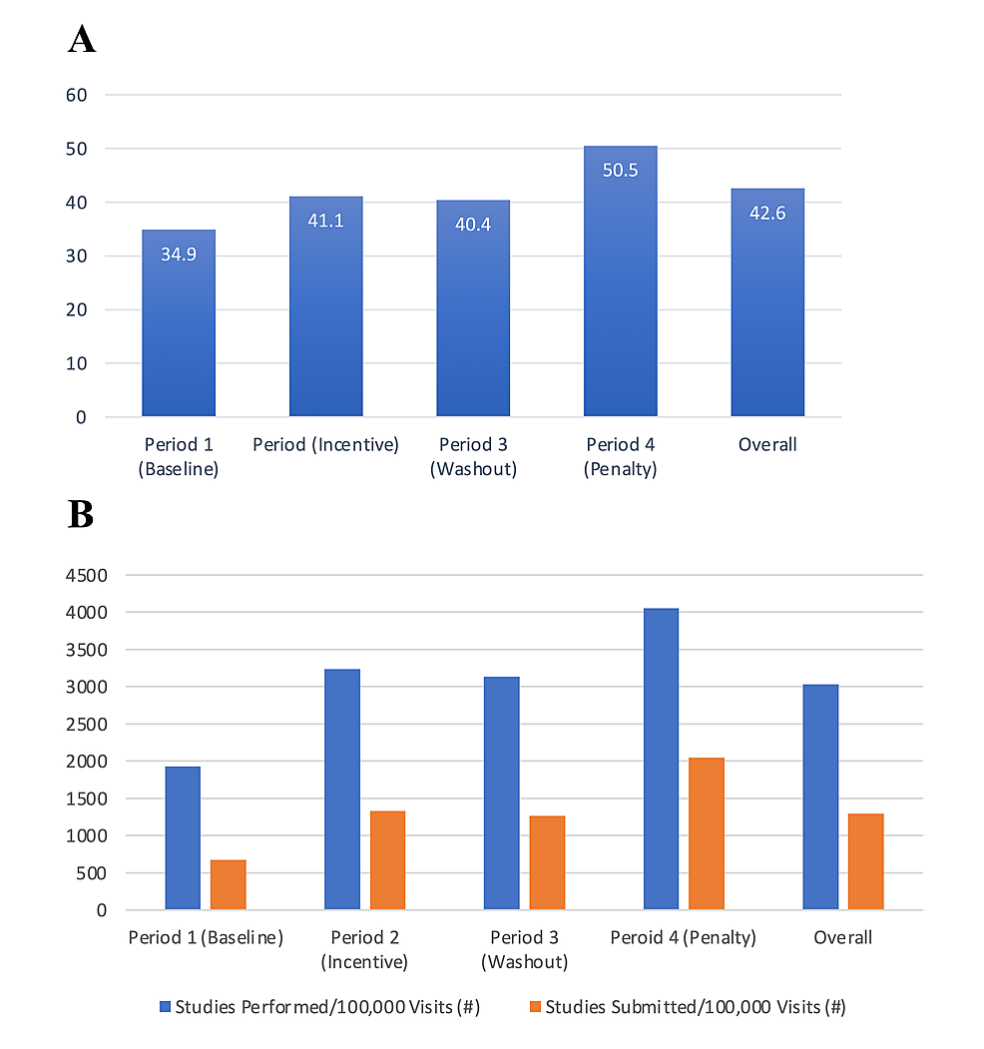
Completion of workflow documentation. A. Percentage of point-of-care ultrasound studies submitted in each time period. B. Studies performance and submission per 100,000 patients in each time period.

Figure 4 reveals completion of workflow documentation rate - the percentage of studies submitted per studies performed - for all providers for each time period. These data show the same pattern as Figure 2: The Incentive and Penalty time periods showed improvements with relative stability seen during the Washout period. Figure 4 also reveals that this same pattern was seen not only in the number of studies submitted, but also in the volume of studies performed when adjusted to show the expected numbers in an ED that sees 100,000 patients per year.

Table 2 provides the data visualized in Figure 4 but includes a further analysis of the 40 providers. These providers were split into quartiles based on the number of patients seen overall during the study period, ranked from low to high. Thus, the first quartile includes the 10 providers who saw the fewest number of patients during the study period, with each successive quartile containing a set of 10 providers with the next highest overall patient volume. By sorting the data in this way, it became clear that, overall, the number of studies performed per 100,000 ED patient visits was relatively stable during the study period at around 3000 studies per 100,000 patients (3%). That said, the overall percentage of studies submitted (completed workflow) increased with each quartile from low to high. In this data set, each quartile had similar rates of ultrasounds performed, but there was a clear trend toward higher rates of POCUS workflow completion among the providers who saw more patients overall during the study period.

**Table 2:** Numerical data for the percentage of POCUS studies submitted, as well as the number of studies performed and submitted (adjusted for an ED that sees 100,000 patients per year) in each time period for all 40 providers in addition to quartiles of 10 providers each based on number of patients seen during the entire study period. POCUS, point-of-care ultrasound; ED, emergency department.

	Period 1 (Baseline)	Period 2 (Incentive)	Period 3 (Washout)	Period 4 (Penalty)		Overall
All Providers						
Submitted/Performed (%)	34.9	41.1	40.4	50.5	42.6	
Performed/100,000 Visits	1927	3233	3133	4052	3034	
Submitted/100,000 Visits	673	1330	1265	2046	1293	
First Quartile (Low)						
Submitted/Performed (%)	17.5	13.5	27.6	26.5	21.7	
Performed/100,000 Visits	2093	3390	3974	3790	3217	
Submitted/100,000 Visits	367	458	1096	1003	697	
Second Quartile						
Submitted/Performed (%)	35.1	29.7	34.4	46.7	36.6	
Performed/100,000 Visits	2032	2833	3153	3422	2805	
Submitted/100,000 Visits	712	840	1084	1599	1026	
ThirdQuartile						
Submitted/Performed (%)	33.1	35.9	37.1	47.2	39	
Performed/100,000 Visits	2142	3250	3092	4162	3129	
Submitted/100,000 Visits	708	1168	1146	1966	1221	
Fourth Quartile (High)						
Submitted/Performed (%)	43.2	56.4	50.4	59	53.8	
Performed/100,000 Visits	1680	3377	2931	4344	3036	
Submitted/100,000 Visits	725	1906	1477	2561	1635	

## Discussion

The results of this study reveal a positive association between the implementation of both positive and negative financial incentives and POCUS documentation rate among the attending physicians at an academic ED. In other words, both the incentive and the penalty were associated with similar increases in physician documentation workflow completion. A potential concern with physician performance incentives is over-testing or unnecessary testing. But these data suggest that the use of ultrasound was not increased, rather the completion of the workflow increased. Data showing that positive and negative incentives equally influence physician workflow completion may help guide physician leaders in designing workflow incentives at other institutions. These results may aid future research on physician documentation incentives. 

When the study participants were split into quartiles based on the overall number of patients seen during the study period, additional interesting trends were noted. These data revealed that all quartiles had similar rates of ultrasound performance (per 100,000 ED visits) at around 3% but displayed a trend toward higher rates of POCUS documentation among those faculty attending physicians that cared for more patients per shift. In fact, the difference in overall documentation rate between the first and fourth quartiles was 21.7% versus 53.8%. In other words, the providers that were more productive in terms of patients seen per shift were much more likely to complete the ultrasound workflow documentation. This higher rate of documentation could be related to greater comfort with the workflow among the providers who saw more patients over the study period or another undetermined factor. Although this study was not able to delineate why those providers who saw more patients overall also documented ultrasounds with higher frequency, it did reveal that all quartiles utilized POCUS with similar frequency (normalized by patient volume). Each group was performing ultrasounds at similar rates, but the providers evaluating more patients were more likely to submit the ultrasounds in all study periods. These data can be useful to emergency ultrasound directors as there may be benefit in focused education on efficient ultrasound documentation workflows. 

This study’s main results are only adjusted for one factor (i.e. number of patients seen per month) and are, therefore, subject to other sources of confounding. One example of this might be the impact of resident physicians. Attending physicians evaluate a vast majority of their patients alongside resident physicians and are randomly paired with residents. At our institution, the attending and resident physicians are scheduled in a manner that is not expected to lead to a heightened frequency of any particular attending-resident pairing. Over each study period, any individual resident influences (e.g. a senior resident with interest in advanced ultrasound training after residency) would be equally dispersed among the entire attending group.

An additional limitation of this study is its constricted generalizability, as it was only performed at a single site. It should be noted, however, that an American College of Emergency Physicians’ Round Table poll revealed that Qpath and Epic were the most frequently utilized POCUS archival and EHR programs, respectively, among the participating institutions [10]. The findings of this poll, therefore, enhance the scalability of the study results from our single institution.

Finally, it is not possible to separate the financial portion of the incentive in this study from the very fact that an incentive was being offered in the first place. Physicians would know that their institution places a high priority on POCUS documentation if it is willing to augment or withhold bonuses. This prioritization serves as a signpost and may in and of itself motivate the physicians (independent of money).

Further research in this field may focus on determining whether the size of the monetary incentive matters and whether financial incentives are superior to other interventions (e.g. focused education programs, automated reminder emails, real-time feedback, or further streamlining the POCUS documentation workflow).

## Conclusions

This study reveals a positive association between the implementation of both financial incentives and financial penalties with increases in POCUS documentation among attending physicians at an academic ED. Improvement in workflow completion was seen across a large portion of the providers. In this dataset, providers caring for the more patients per shift also completed the workflow a greater percentage of the time in all time periods.
